# Association between periodontitis, leukoaraiosis and sTWEAK. A case-control study

**DOI:** 10.3389/froh.2026.1737799

**Published:** 2026-03-03

**Authors:** Jorge Moldes, Antonio Arnau, Manuel Rodríguez-Yáñez, Iria López-Dequidt, Pablo Hervella, Juan Blanco, Ramón Iglesias-Rey, Tomás Sobrino, Yago Leira

**Affiliations:** 1 Family and Community Medicine Department, Bueu Health Centre, Pontevedra University Clinical Hospital, Pontevedra, Spain; 2 Periodontology Unit, Faculty of Odontology and Medicine, University of Santiago de Compostela, Santiago de Compostela, Spain; 3 Stroke Unit, Neurology Department, University Clinical Hospital, Santiago de Compostela, Spain; 4 Stroke Unit, Neurology Department, University Clinical Hospital, A Coruña, Spain; 5 Neuroimaging and Biotechnology Laboratory (NOBEL) Group, Clinical Neurosciences Research Laboratories, Health Research Institute of Santiago de Compostela, University Clinical Hospital, Santiago de Compostela, Spain; 6 Network Center for Biomedical Research in Neurodegenerative Diseases (CIBERNED), Institute of Health Carlos III, Madrid, Spain; 7 NeuroAging Group, Clinical Neurosciences Research Laboratories, Health Research Institute of Santiago de Compostela, University Clinical Hospital, Santiago de Compostela, Spain

**Keywords:** cerebral small vessel disease, leukoaraiosis, neurology, periodontitis, risk, sTWEAK

## Abstract

**Background:**

In recent years, periodontitis has been associated with various systemic diseases, including neurological pathology, and systemic inflammation has been proposed as a potential link. The aim of this study was to investigate the relationship between periodontitis and the presence of leukoaraiosis (LA). The contribution of periodontitis to serum levels of soluble fragment of tumor necrosis factor-like weak inducer of apoptosis (sTWEAK) in LA was also explored.

**Methods:**

We performed a nested case-control study including patients with LA (*n* = 70) and control subjects without known neurological diseases (*n* = 140). A full-mouth periodontal examination was carried out in all participants. Socio-demographic and oral care related data, self-reported vascular risk factors and body mass index (BMI) were also recorded. Neuroimaging and ultrasonographic assessments were performed in LA patients. Blood samples were collected from all participants to determine serum sTWEAK levels by enzyme-linked immunosorbent assay technique.

**Results:**

Both the prevalence of periodontitis and mean serum sTWEAK concentrations were significantly higher in LA patients than controls (74.3% vs. 32.1%, *p* < 0.001 and 247.5 ± 90.2 vs. 27.5 ± 17.6 pg/mL, *p* < 0.001). With multiple logistic regression analysis, a positive relationship was shown between periodontitis and presence of LA independent of well-known LA risk factors such as age, gender, hypertension, diabetes, dyslipidemia and BMI (OR = 6.3; 95% CI: 2.5–15.6, *p* < 0.001). In LA cases, periodontitis was associated with increased sTWEAK levels (B coefficient = 198.3; 95%CI: 95.5–301.2, *p* < 0.001).

**Conclusions:**

Periodontitis may be associated with the presence of LA and when present it could contribute to elevated serum levels of sTWEAK.

## 1 Introduction

Leukoaraiosis (LA) is a radiological term that refers to cerebral white matter abnormalities/Lesions observable as signal hypodensities on computed tomography (CT) scans of hyperintensities on T2-weighted magnetic resonance imaging (MRI) scans (1–3). The prevalence of LA is high worldwide among middle-aged and is becoming a significant public health burden due to the rapid increase in the aging population (4). Asymptomatic LA is closely linked to a pathological evolution to cerebral small vessel disease (cSVD) (5) which, in turn, is the prelude of more severe neurological diseases such as early dementia and Alzheimer's disease (6, 7) or stroke (8–13). Moderate-to-severe LA cases have shown to suffer from vascular endothelial dysfunction (14) and blood brain barrier damage (15). Recently, our group identified an endothelial dysfunction biomarker, the soluble tumor necrosis factor-like inducer of apoptosis (sTWEAK), as a possible circulating marker related to LA presence in ischemic stroke patients (16).

Periodontitis not only induces a local immune-inflammatory reaction in the gingival tissues but also negatively affects overall's health. In the case of neurological diseases, periodontitis has been associated with increased risk of developing cSVD [i.e., lacunar infarct (LI)] (17), ischemic stroke (18) and Alzheimer's disease (19). Recent preclinical data has shown that peripheral levels of sTWEAK increases after induction of experimental periodontitis (20). In addition, our group observed that periodontitis was associated with high serum sTWEAK levels in LI patients (21). Therefore, the potential ability of periodontitis to produce systemic elevation of sTWEAK concentrations may represent a plausible biological mechanism behind the link with the presence of LA.

The aim of this study was two-fold: to compare the prevalence of periodontitis in patients with LA with a group of control subjects without known cerebrovascular disease; and second, to determine whether the presence of periodontitis was associated with higher sTWEAK levels in the LA cases.

We hypothesized that both the prevalence and severity of periodontitis would be higher in the LA group and that greater periodontal severity would correlate with increased sTWEAK concentrations among LA patients.

## 2 Materials and methods

### 2.1 Study design and participants

We designed a case-control study including subjects recruited at Stroke Unit of University Clinical Hospital of Santiago and two primary care centres in Galicia (Spain) between the years 2014 and 2019. The design employed in this study has already been described in a related publication (17). Cases were those with a diagnosis of leukoaraiosis defined according to Hachinski and co-workers as bilateral and symmetrical areas in the periventricular and centrum semiovale white matter that appears hypodense on CT scans and hyperintense on T2-weighted MRI (3). Cases were included in the study if they fulfilled the following inclusion criteria: (a) >18 years of age; (b) at least 15 teeth (excluding third molars); and (c) written informed consent (17). Exclusion criteria were as follows: (a) patient who have received periodontal treatment in the previous 12 months; (b) systemic antibiotics, corticosteroids, and/or immunosuppressant therapy within 3 months prior to periodontal assessment; and (c) chronic use of non-steroidal anti-inflammatory drugs (17).

Recruitment of control group was published elsewhere (17). In brief, healthy control subjects were selected from the hospital database. In order to include individuals without any neurological disorder, we reviewed 194 CT/MRI scans of subjects who were referred to the Department of Neurology with a suspicious diagnosis of non-confirmed neurological diseases such as non-specific headache, vestibular syndromes, brain tumours or altered level of consciousness between 2009 and 2013 (17). Of these, 12 presented some subtype of asymptomatic cSVD [silent infarcts (SI), *N* = 4; LA, *N* = 8] and, thus, were excluded from the study. Therefore, 182 subjects free from any neurological disease were contacted by telephone and asked to participate. Inclusion and exclusion criteria were the same as for the case group. Control individuals were clinically examined and interviewed in parallel with patient recruitment. For both cases and controls, demographic and medical information were obtained by means of a questionnaire (17).

The present study was performed in accordance with the Declaration of Helsinki of the World Medical Association (2008) and approved by the Ethics Committee of Santiago-Lugo (ID: 2016/399) (17). Informed consent was obtained from each patient or their relatives after full explanation of the procedures. The study followed the STROBE Guidelines for observational human research (22).

The sample size calculation was performed using the Macro! NSize for PASW Statistics (http://www.metodo.uab.cat/macros.htm.). Based on a pilot analysis, to detect an expected odds ratio (OR) of 3.0 in the association between periodontitis and LA, and assuming α-risk = 0.05 and β-risk = 0.15, a sample of 210 subjects was calculated (70 cases and 140 controls, 1:2 case:control) (17).

### 2.2 Study protocols (neuroimaging, ulstrasonographic and oral assessment, and laboratory analysis)

The methodology used for neuroimaging, ultrasonographic and oral examinations, as well as for biochemical analysis of sTWEAK serum levels, has been thoroughly described in previous publications by our research group (17, 21). As the procedures applied in the present study are identical to those previously reported, a detailed description is provided in the Supplementary Material.

### 2.3 Other covariates

Socio-demographic data recorded used in the present analysis included age and gender and educational level (low was defined as those participants who dropped out of school before age 14). Body weight was measured to the nearest 1 kg, and height was recorded to the nearest centimetre. Body mass index (BMI) was calculated with the formula weight (kg)/height (m)2. Cardiovascular risk factors (CVRF) were recorded: previous history of smoking (current smoker), alcohol consumption (considering heavy drinker as >300 g of alcohol/week), history of diabetes [glycated haemoglobin ≥6.5%, glycaemia ≥200 mg/dL in symptomatic patients, baseline glycaemia ≥126 mg/dL in 2 determinations or glycaemia after oral glucose tolerance test ≥200 mg/dL or under anti-diabetic medication], hypertension (blood pressure ≥140/90 mmHg in 2 determinations or under anti-hypertensive medication) and hypercholesterolemia [total cholesterol >250 mg/dL or low-density lipoprotein cholesterol >130 mg/dL or under lipid-lowering medication].

### 2.4 Statistics

The statistical methods were previously published in another study by our group (17), with specific adaptations made to address the objectives of the present analysis.

All data analyses were performed with IBM SPSS Statistics 20.0 software for Mac (SPSS Inc., Chicago, IL, USA). Continuous normally distributed variables analysed with Kolmogorov–Smirnov test were reported as mean ± standard deviation, whereas categorical variables were expressed as percentages. Differences between two groups were assessed by independent t test (continuous normally distributed variables) and *χ*^2^ test (categorical variables). Non-normally distributed variables were showed as median (interquartile range) and compared using Mann–Whitney *U* test. Differences between more than two groups were tested by one-way ANOVA. Conditional logistic regression models were performed to test potential associations between periodontitis and its clinical parameters and LA presence. Multivariable linear regression analysis was done to investigate the contribution of periodontitis and its clinical parameters to increased serum sTWEAK levels.

All tests were performed at a significance level of *α* = 0.05.

## 3 Results

### 3.1 Study groups – baseline characteristics

Table 1 shows baseline characteristics of LA cases and controls. Patients with LA were older than controls. The number of females was lower in the control group compared with cases. Participants with LA had more frequently a previous history of hypertension, diabetes, dyslipidemia as well as higher BMI values than those subjects without LA. No statistically significant differences were observed between study groups for smoking habit, alcohol consumption or education level.

**Table 1:** Baseline characteristics of LA cases and controls.

Variables	Cases (*n* = 70)	Controls (*n* = 140)	*P*-value
Age (years)	70.9 ± 5.7	65.2 ± 10.2	<0.001
Females, *n* (%)	31 (44.3)	36 (25.7)	0.006
Arterial hypertension, *n* (%)	61 (87.1)	40 (28.6)	<0.001
Diabetes mellitus, *n* (%)	30 (42.9)	12 (8.6)	<0.001
Dyslipidemia, *n* (%)	40 (57.1)	25 (17.9)	<0.001
Current smoker, *n* (%)	13 (18.6)	17 (12.1)	0.430
Heavy drinker, *n* (%)	6 (8.6)	7 (5.0)	0.590
Low education level, *n* (%)	31 (44.3)	46 (32.9)	0.187
BMI	29.4 ± 4.7	26.5 ± 4.5	<0.001

BMI, body mass index.

### 3.2 Study groups—periodontitis

**Figure 1:**
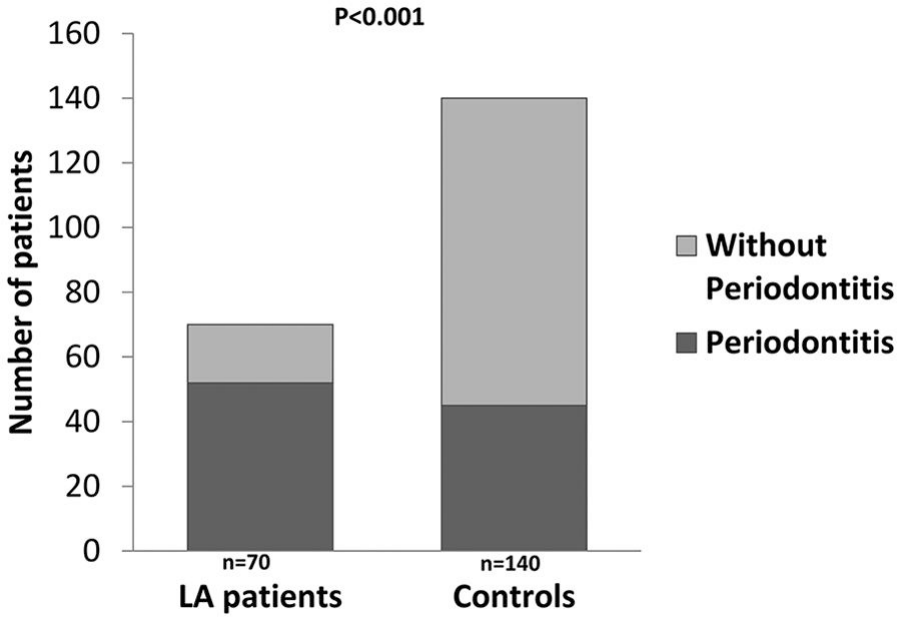
Prevalence of periodontitis in patients with and without LA.

**Figure 2:**
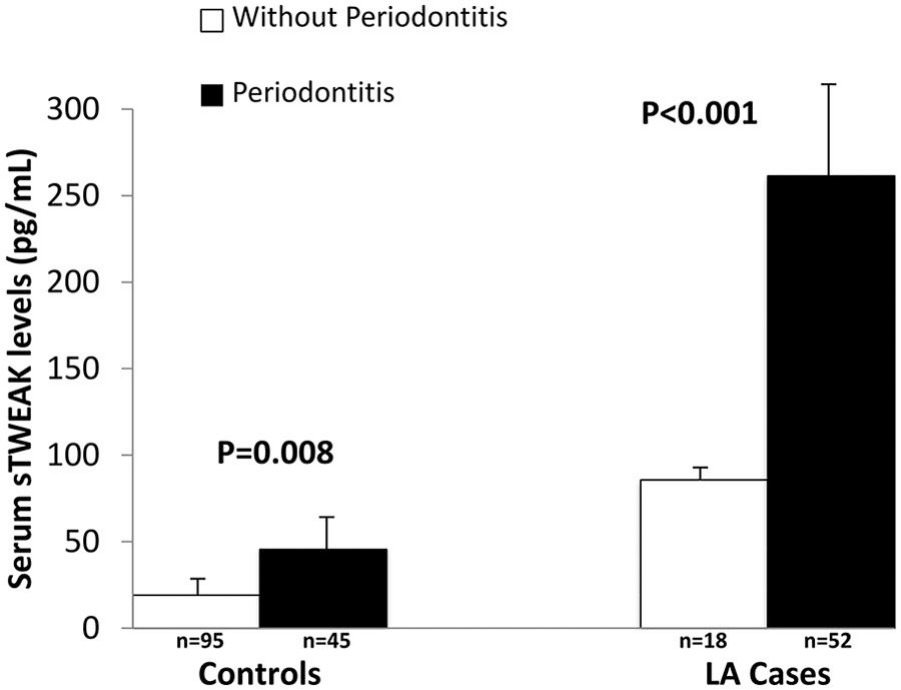
Percentage of patients according to periodontitis severity in participants with and without LA.

LA patients had worse periodontal conditions when compared to controls. Comparisons of clinical periodontal parameters between study groups are shown in Table 2. Accordingly, periodontitis was present in 45 of 140 control subjects (32.1%) and in 52 of 70 patients with LA (74.3%). This almost 2.5-fold increase in the proportion of patients with LA who had periodontitis compared with control subjects was highly significant (*p* < 0.001) (Figure 1). The prevalence of periodontitis in 32.1% of control subjects is consistent with the prevalence observed in the Spanish adult population (38%) (23). With regards to periodontitis severity, half of the LA patients with periodontitis had severe periodontitis compared to 6.7% in the control group (Figure 2). No differences between study groups were found for oral care related variables (Table 2).

**Table 2:** Periodontal parameters and oral care-related variables of LA cases and controls.

Variables	Cases (*n* = 70)	Controls (*n* = 140)	*P*-value
FMPS (%)	51.2 ± 21.8	27.6 ± 12.6	<0.001
FMBS (%)	55.1 ± 22.4	28.7 ± 14.2	<0.001
PD (mm)	3.7 ± 1.3	2.6 ± 0.7	<0.001
Sites with PD ≥6 mm (%)	8.0 [0.0, 30.7]	0.0 [0.0, 0.0]	<0.001
CAL (mm)	4.3 ± 1.7	3.0 ± 1.0	<0.001
Sites with CAL ≥5 mm (%)	17.5 [0.0, 85.0]	2.0 [1.0, 13.0]	0.001
Number of present teeth	21.0 [18.0, 24.0]	26.0 [23.0, 27.0]	<0.001
PISA (mm^2^)	1,036.7 ± 1,073.3	187.5 ± 345.0	<0.001
Last dental visit >12 months, *n* (%)	21 (36.8)	45 (32.1)	0.526
Tooth brushing <2 times/day, *n* (%)	15 (26.3)	45 (32.1)	0.420
Use of interdental care devices, *n* (%)	7 (12.3)	15 (10.7)	0.752

FMPS, full-mouth plaque score; FMBS, full-mouth bleeding score; PD, probing pocket depth; CAL, clinical attachment level; PISA, periodontal inflamed surface area.

The prevalence of LIs in LA patients was significantly higher in those with periodontitis than in LA patients without periodontitis (76.9% vs. 33.3%, *p* < 0.001). However, no major differences were noted neither for the presence of subclinical atherosclerosis (*p* = 0.275) nor for silent infarcts (*p* = 0.124). With regards to the degree of LA severity, patients with and without periodontitis had a similar prevalence of LA degree II-III (82.7% vs. 89.9%, *p* = 0.690).

### 3.3 Association of periodontitis and its clinical parameters with LA

Multiple logistic regression analysis was performed to assess the association between periodontitis and LA, adjusting for age, sex, hypertension, diabetes, dyslipidemia, and BMI. The results are summarized in Table 3.

**Table 3:** Multivariable logistic regression analysis of the association between periodontitis and leukoaraiosis.

Exposure variables	OR	95% CI	*P*-value
Periodontitis (yes vs. no)	6.3	2.5–15.6	<0.001
Severe periodontitis (vs. no/mild)	26.4	5.6–235.1	<0.001
Mean PD (per 1 mm increase)	3.9	2.3–6.8	<0.001
Mean CAL (per 1 mm increase)	2.6	1.7–3.8	<0.001
PISA (per 1 mm^2^ increase)	1.003	1.002–1.004	<0.001

OR, odds ratio; CI, confidence interval; PD, probing pocket depth; CAL, clinical attachment level; PISA, periodontal inflamed surface area. All models were adjusted for age, sex, hypertension, diabetes mellitus, dyslipidemia, and body mass index.

Periodontitis was significantly associated with the presence of LA (OR = 6.3; 95% CI: 2.5–15.6, *p* < 0.001), and a stronger association was observed for severe periodontitis (OR = 26.4; 95% CI: 5.6–235.1, *p* < 0.001). These associations remained consistent when continuous measures of periodontal disease were considered in the adjusted models: probing depth (PD) (OR = 3.9; 95% CI: 2.3–6.8, *p* < 0.001), clinical attachment level (CAL) (OR = 2.6; 95% CI: 1.7–3.8, *p* < 0.001) and periodontal inflamed surface area (PISA) (OR = 1.003; 95% CI: 1.002–1.004, *p* < 0.001).

### 3.4 sTWEAK

**Figure 3:**
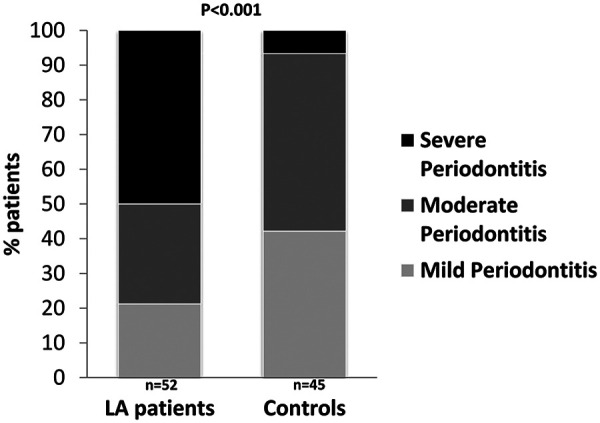
sTWEAK serum levels according to periodontal status in cases and controls.

Patients with LA had significantly higher mean serum sTWEAK levels than controls (247.5 ± 90.2 vs. 27.5 ± 17.6 pg/mL, *p* < 0.001). Figure 3 depicts that the presence of periodontitis was associated with higher mean sTWEAK concentrations in both groups.

Multivariable linear regression models adjusted for age, gender, hypertension, diabetes, dyslipidemia and BMI were carried out in the 70 patients with LA. Results showed that mean serum sTWEAK levels were significantly and positively associated with greater mean PD (B coefficient = 58.7; 95%CI: 36.7–80.7, *p* < 0.001), CAL (B coefficient = 39.3; 95%CI: 23.2–55.4, *p* < 0.001) and PISA (B coefficient = 0.06; 95%CI: 0.04–0.08, *p* < 0.001). When periodontitis exposure was examined as a categorical variable, multivariable linear regression analysis again indicated that the presence of periodontitis in patients with LA was associated with significantly higher serum sTWEAK concentrations (B coefficient = 198.3; 95%CI: 95.5–301.2, *p* < 0.001).

## 4 Discussion

In our study, we observed that almost 3/4 of our patients with LA were diagnosed with periodontitis. When present, periodontitis emerged as a significant and independent contributor to increased systemic sTWEAK concentrations.

Our findings reveal a statistically significant association between periodontitis and its clinical parameters [PD, CAL, PISA, full-mouth plaque score (FMPS) and full-mouth bleeding score (FMBS)] with LA, in a model adjusted for multiple CVRF. Moreover, a stronger association was observed between severe periodontitis and LA. Previous scientific evidence shows considerable heterogeneity in terms of methodology, magnitude, and significance of the association; however, it tends to support a link between these two conditions. In 2019, our group had already reported a higher prevalence of LA in patients with periodontitis and LI using a model adjusted for multiple covariates (17). A more recent analysis (24) found that LA is correlated with periodontal and oral health variables [CAL, plaque index and decayed, missing and filled teeth index (DMFT)]; however, this association was lost after adjusting for CVRF. Nevertheless, the analysis of white matter microstructural integrity using peak width of skeletonized mean diffusivity (PSMD)—a more sensitive marker of subtle brain damage—maintained a significant association with DMFT and plaque index even after full adjustment. Other studies used tooth loss as indicator of periodontal status (25, 26), also reporting an association between these conditions. However, results from multivariate analyses were inconsistent: the association persisted for severe tooth loss in one study (25) but disappeared in another after adjusting for age (26). Variability in findings may stem from methodological differences, such as the use of CT instead of MRI to identify LA, or reliance on composite outcomes (25). Additionally, tooth count may be not an accurate indicator of periodontal disease, as it does not clarify the cause of tooth loss (25, 26). In this context, our study contributes to clarifying the existing controversy and supports the existence of an association between periodontitis and LA. Nevertheless, further well-designed prospective studies are warranted to investigate this relationship.

Other manifestations of cSVD were also evaluated in this study. A higher prevalence of LI was observed in subjects with both LA and periodontitis compared to those without periodontal disease, whereas no differences were found in the prevalence of SI. Previous publications from our group (17, 27) reported an independent association between clinical periodontitis and LI, regardless of multiple known CVRF. Furthermore, the subgroup with severe periodontitis showed a stronger association (17). However, findings from the Atherosclerosis Risk in Communities (ARIC) cohort study (28) did not identify any relationship. These discrepancies may be attributed to methodological differences, including study design, population, definition of lacunar infarct, or the covariate adjustment. Despite these inconsistencies, the overall body of evidence appears to support an association between both conditions. SI have been previously linked to periodontitis in several studies. In 2013, a study in a Japanese population reported a trend toward an association between probing depth and SI, although the association did not remain significant in multivariable analysis (29). That same year, another study suggested a potential link through a combined analysis of SI and LA in patients with severe tooth loss (25). Using a different approach (30) an association was found between SI and occlusal support assessed by CT, a metric that reflects not only tooth number but also functional oral integrity. From a radiological standpoint, two studies also demonstrated a relationship between alveolar bone loss and the incidence of SI (29, 30). In contrast, studies from our group did not observe any association (17, 27), and a recent analysis from the ARIC cohort (31) even reported an inverse relationship between periodontitis and SI. These discrepancies may be explained by limitations such as insufficient sample size to assess SIs (17, 27), as well as potential biases in selection, attrition, and information (31). Considering the overall body of evidence and the fact that assessing SI was not the primary aim of our study, it is possible that our study design and sample size were not optimal for evaluating this outcome.

The second aim of our study was to evaluate plasma levels of sTWEAK in patients with periodontitis and LA. This molecule belongs to the TNF superfamily and is secreted by cells of the mononuclear-phagocyte system. It functions as an inflammatory mediator through its receptor Fn14, promoting the release of pro-inflammatory cytokines [such as interleukin (IL)-8 and intracellular adhesion molecule (ICAM)-1 and vascular adhesion molecule (VCAM)-1] (32, 33). Additionally, sTWEAK promotes the production of matrix metalloproteinases and contributes to the disruption of the blood–brain barrier (34, 35). Previous studies have demonstrated an association between TWEAK and periodontitis both in its tissue form (32, 33, 36) and in its soluble form (20, 21). Similarly, other studies have linked elevated sTWEAK levels with the presence and severity of LA in patients with intracerebral haemorrhage (16, 37), ischemic stroke (38, 39), and LI (16). In our study, we observed that sTWEAK levels were elevated independently in patients with periodontitis and in those with LA, reaching their highest levels in patients presenting both conditions. Furthermore, clinical parameters of periodontitis (PD, CAL, PISA) were independently associated with increased sTWEAK levels. These findings align with previous studies showing an association between TWEAK and periodontitis (20, 21, 32, 33, 36) and between elevated sTWEAK levels and LA in cerebrovascular diseases (16, 37–39), supporting the hypothesis that sTWEAK may serve as a mechanistic link between these two clinical entities.

Taken together, our findings confirm our study hypothesis: both the prevalence and severity of periodontitis were higher in patients with LA, and greater periodontal severity was associated with increased circulating sTWEAK levels among LA patients. These results support the notion that sTWEAK may represent a mechanistic link between periodontitis and LA.

The main strengths of this article include the unbiased screening of individuals, the presence of a well-defined control group, appropriate adjustment for CVRF, and the combined use of qualitative and continuous quantitative variables. However, there are several limitations to our study. First, it was conducted at a single centre, which limits the external validity. Second, due to the retrospective case-control design, causal or unidirectional relationships between periodontitis and LA cannot be established. Third, controls were selected from hospital-based databases and underwent neuroimaging to exclude silent cerebrovascular disease. Nevertheless, this group may not fully represent the general population, and some degree of selection bias cannot be completely ruled out. Fourth, we evaluated only sTWEAK and did not assess its association with Fn14, which may have greater implications in LA pathogenesis. Fifth, serum sTWEAK levels are not specific markers for a particular pathological process. Finally, although multivariable adjustment was performed for major vascular risk factors, residual confounding by unmeasured or incompletely measured variables cannot be excluded; other potential confounders, such as large-vessel atherosclerosis, chronic kidney disease, and hyperhomocysteinemia, were not controlled for. Future prospective and longitudinal studies are warranted to confirm the temporal relationship between periodontitis and LA. Studies incorporating additional inflammatory and endothelial biomarkers, including IL-8, matrix metalloproteinases, and the Fn14 receptor, as well as population-based control groups, would further clarify the underlying biological mechanisms.

## 5 Conclusion

Periodontitis is highly prevalent among LA patients. The presence of periodontitis appeared to contribute to sTWEAK level elevations, an effect that was independent of well-known contributing factors.

## Data Availability

The raw data supporting the conclusions of this article will be made available by the authors, without undue reservation.
