# Supplementary material for: Association between periodontitis, leukoaraiosis and sTWEAK. A case-control study
Source: Front Oral Health. 2026 Mar 3;7:1737799. doi: 10.3389/froh.2026.1737799 (PMC12992221; doi:10.3389/froh.2026.1737799)
Supplement: Supplementary file 1 [file Datasheet1.docx]

Supplementary Material

# MATERIALS AND METHODS

## Neuroimaging and ultrasonographic examination

Neuroimaging and ultrasonographic assessment were described previously [1]. A CT/MRI scan was carried out in all participants at admission. MRI images were obtained on a 1.5 T system (1.5 Magneton Symphony, Siemens, Erlangen, Germany), with echo planar capabilities of 25 mT/m gradients and 300–350 μs rise times. The MRI protocol included T1-w (TR/TE: 370/7.7 ms), T2-w (TR/TE: 6020/113 ms), DP-w (TR/TE: 6020/113 ms) and FLAIR (TR/TE: 9000/114 ms) [2]. One neuroradiologist who was blinded to the clinical data carried out the evaluation of CT/MRI. LA was defined as ill-defined hyperintensities ≥5 mm on both T2 and FLAIR MRI images without prominent hypointensities on T1-w MRI scans and as ill-defined and moderately hypodense areas of ≥5 mm on CT. LA was classified according to the Fazekas criteria [3,4] using the modified Fazekas scale [5]. This method yields two separate scores for subcortical and deep white matter lesions and periventricular lesions. The four-point Fazekas scale of increasing severity was used to classify each score. Presence of LI was diagnosed if the patient had one of the characteristic clinical lacunar syndromes, neurological deficit lasting >24 hr, no evidence of cerebral cortical dysfunction, and a CT/MRI that showed a deep focal infarction in an appropriate location with a diameter ≤15 mm. The presence of a LI in the baseline CT in which the topography does not correspond with the present clinical syndrome was considered a silent infarct.

To evaluate the existence of carotid atherosclerosis, the carotid intima-media thickness (IMT) was measured as previously described [1]. Briefly, the image was focused on the posterior (far) wall of the left carotid artery. A minimum of four measurements of the common carotid far wall was taken 10 mm proximal to the bifurcation, to derive the mean carotid IMT [6]. The presence of an atheroma plaque was evaluated in the common and internal carotid extracranial arteries as well as the bifurcations according to standardized scanning and reading protocols [7]. The presence of carotid subclinical atherosclerosis was defined as an IMT value >0.89 mm in men and >0.82 mm in women [8].

## Oral examination

Full mouth periodontal assessments were done by a trained periodontist (YL) who was previously calibrated [1]. All measurements were performed using a calibrated University of North Carolina periodontal probe (UNC15, Hu-Friedy, Chicago, IL, USA) at six sites per tooth (excluding third molars). Clinical periodontal parameters recorded included pocket depth (PD), clinical attachment level (CAL), dental plaque accumulation, and gingival bleeding as previously described [1]. The presence of mild periodontitis was established when ≥2 interproximal sites with CAL ≥3 mm and ≥2 interproximal sites with PD ≥4 mm (not on the same tooth) was detected or 1 site with PD ≥5 mm was present. Moderate PD was defined as ≥2 interproximal sites with CAL ≥ 4 mm (not on the same tooth) or ≥2 interproximal sites with PPD ≥ 5 mm, also not on the same tooth. Severe PD was defined as the presence of ≥2 interproximal sites with CAL ≥ 6 mm (not on the same tooth) and ≥1 interproximal site with PPD ≥ 5 mm. Total periodontitis was the sum of mild, moderate, and severe PD [9,10]. Additionally, a measure of periodontitis activity, the periodontal inflamed surface area (PISA), which reflects the surface area of bleeding pocket epithelium in mm2, was calculated [11].

Oral healthcare habits including tooth brushing frequency, use of interdental care devices and frequency of visits to dentist were recorded.

## sTWEAK analysis

Serum analysis methods were previously reported in a related publication by our group [12]. On admission for cases and periodontal examination/interview day for controls, 2 mL of venous blood were collected from the antecubital fossa by venepuncture using a 20-gauge needle with a 2-mL syringe. Blood samples were allowed to clot at room temperature and after 1 hour, serum was separated from blood by centrifugation (15 minutes at 3,000 g) and 0.5 mL of extracted serum was immediately transferred to 1.5-mL aliquots. Each aliquot was stored at –80 °C until the time of analysis. Serum levels of the investigated biomarker were measured by enzyme-linked immunosorbent assay (ELISA) technique following the manufacturer's instructions. sTWEAK ELISA kit (Aviscera Bioscience, Santa Clara, CA) minimum assay sensitivity was 10 pg/mL, with an intra-assay coefficient of variation of 5.0% and inter-assay CV of 2.3%. Determinations were performed in the Clinical Neurosciences Research Laboratory in a blind fashion.

# REFERENCES

1.Leira Y, Rodríguez‐Yáñez M, Arias S, López‐Dequidt I, Campos F, Sobrino T, D'Aiuto F, Castillo J, Blanco J. (2019) Periodontitis as a risk indicator and predictor of poor outcome for lacunar infarct. J. Clin. Periodontol. 46(1):20–30. DOI: 10.1111/jcpe.13032.

2.Rodríguez I, Lema I, Blanco M, Rodríguez-Yáñez M, Leira R, Castillo J. (2010) Vascular retinal, neuroimaging and ultrasonographic markers of lacunar infarcts. Int. J. Stroke 5(5):360–366. DOI: 10.1111/j.1747-4949.2010.00462.x.

3.Fazekas F, Kleinert R, Offenbacher H, Payer F, Schmidt R, Kleinert G, Radner H, Lechner H. (1991) The morphologic correlate of incidental punctate white matter hyperintensities on MR images. Am. J. Neuroradiol. 12(5):915–921.

4.Fazekas F, Kleinert R, Offenbacher H, Schmidt R, Kleinert G, Payer F, Radner H, Lechner H. (1993) Pathologic correlates of incidental MRI white matter signal hyperintensities. Neurology 43(9):1683. DOI: 10.1212/wnl.43.9.1683.

5.Pantoni L, Simoni M, Pracucci G, Schmidt R, Barkhof F, Inzitari D. (2002) Visual rating scales for age-related white matter changes (leukoaraiosis): can the heterogeneity be reduced? Stroke 33(12):2827–2833. DOI: 10.1161/01.STR.0000038424.70926.5E.

6.Raitakari O, Juonala M, Kähönen M, Taittonen L, Laitinen T, Mäki-Torkko N, Järvisalo M, Uhari M, Jokinen E, Rönnemaa T, Åkerblom H, Viikari J. (2003) Cardiovascular risk factors in childhood and carotid artery intima-media thickness in adulthood: The Cardiovascular Risk in Young Finns Study. JAMA 290(17):2277–2283. DOI: 10.1001/jama.290.17.2277.

7.Touboul P, Hennerici M, Meairs S, Adams H, Amarenco P, Bornstein N, Csiba L, Desvarieux M, Ebrahim S, Hernandez R, Jaff M, Kownator S, Naqvi T, Prati P, Rundek T, Sitzer M, Schminke U, Tardif J, Taylor A, Vicaut E, Woo K. (2012) Mannheim carotid intima-media thickness and plaque consensus (2004–2006–2011): an update on behalf of the Advisory Board of the 3rd and 4th Watching the Risk Symposium 13th and 15th European Stroke Conferences, Mannheim, Germany, 2004, and Brussels, Belgium, 2006. Cerebrovasc. Dis. 34(4):290–296. DOI: 10.1159/000343145.

8.Junyent M, Gilabert R, Núñez I, Corbella E, Vela M, Zambón D, Ros E. (2005) Carotid ultrasound in the assessment of preclinical atherosclerosis. Distribution of intima-media thickness values and plaque frequency in a Spanish community cohort. Med. Clin. 125(20):770–774. DOI: 10.1016/S0025-7753(05)72186-2.

9.Eke P, Page R, Wei L, Thornton‐Evans G, Genco R. (2012) Update of the Case Definitions for Population‐Based Surveillance of Periodontitis. J. Periodontol. 83(12):1449–1454. DOI: 10.1902/jop.2012.110664.

10.Holtfreter B, Albandar J, Dietrich T, Dye B, Eaton K, Eke P, Papapanou P, Kocher T. (2015) Standards for reporting chronic periodontitis prevalence and severity in epidemiologic studies. J. Clin. Periodontol. 42(5):407–412. DOI: 10.1111/jcpe.12392.

11.Nesse W, Abbas F, Van Der Ploeg I, Spijkervet F, Dijkstra P, Vissink A. (2008) Periodontal inflamed surface area: quantifying inflammatory burden. J. Clin. Periodontol. 35(8):668–673. DOI: 10.1111/j.1600-051X.2008.01249.x.

12.Leira Y, Rodríguez‐Yáñez M, Arias S, López‐Dequidt I, Campos F, Sobrino T, D'Aiuto F, Castillo J, Blanco J. (2019) Periodontitis is associated with systemic inflammation and vascular endothelial dysfunction in patients with lacunar infarct. J. Periodontol. 90(5):465–474. DOI: 10.1002/JPER.18-0560.
